# Principles of clinical genetics for rheumatologists: clinical indications and interpretation of broad-based genetic testing

**DOI:** 10.1186/s42358-024-00400-z

**Published:** 2024-08-14

**Authors:** Renan Rodrigues Neves Ribeiro do Nascimento, Caio Robledo D’Angioli Costa Quaio, Christine Hsiaoyun Chung, Dewton de Moraes Vasconcelos, Flavio Roberto Sztajnbok, Nilton Salles Rosa Neto, Sandro Félix Perazzio

**Affiliations:** 1 Disciplina de Reumatologia, Universidade Federal de Sao Paulo, Escola Paulista de Medicina, Rua Otonis, 863, Sao Paulo, SP 04025-002 Brazil; 2 Universidade de Sao Paulo Instituto da Crianca (USP Icr), Sao Paulo, Brazil; 3 Fleury Medicina e Saude, Sao Paulo, Brazil; 4 Universidade de Sao Paulo Faculdade de Medicina (USP FM), Sao Paulo, Brazil; 5 Universidade Federal do Rio de Janeiro, Rio de Janeiro, Brazil; 6 Universidade de Santo Amaro (UNISA), Sao Paulo, Brazil

**Keywords:** DNA sequencing technologies, Molecular diagnosis, Genetic tests, Immune-mediated rheumatic diseases, Primary immune regulatory disease

## Abstract

Advances in DNA sequencing technologies, especially next-generation sequencing (NGS), which is the basis for whole-exome sequencing (WES) and whole-genome sequencing (WGS), have profoundly transformed immune-mediated rheumatic disease diagnosis. Recently, substantial cost reductions have facilitated access to these diagnostic tools, expanded the capacity of molecular diagnostics and enabled the pursuit of precision medicine in rheumatology. Understanding the fundamental principles of genetics and diversity in genetic variant classification is a crucial milestone in rheumatology. However, despite the growing availability of DNA sequencing platforms, a significant number of autoinflammatory diseases (AIDs), neuromuscular disorders, hereditary collagen diseases, and monogenic bone diseases remain unsolved, and variants of uncertain significance (VUS) pose a formidable challenge to addressing these unmet needs in the coming decades. This article aims to provide an overview of the clinical indications and interpretation of comprehensive genetic testing in the medical field, addressing the related complexities and implications.

## Background

Advances in DNA sequencing technologies precipitated by next-generation sequencing (NGS) have led to a revolution in the diagnosis of immune-mediated rheumatic diseases. This progress has not only facilitated the adoption of whole-exome sequencing (WES) in clinical practice but also made whole-genome sequencing (WGS) feasible for molecular investigation. With the success of the Human Genome Project, various molecular diagnostic tools have emerged, and the field has experienced exponential growth, enabling the pursuit of precision medicine [1]. Moreover, NGS has become more affordable, and therefore readily available, for both basic researchers and clinicians in recent years [2]. Among myopathies, genetic testing has redefined previous diagnoses of polymyositis due to the increased capability of identifying metabolic myopathies or muscular dystrophies [3]. Similarly, NGS has also allowed for the elucidation of congenital bone diseases and the identification of heritable connective tissue disorders [4, 5]. As access to genetic testing has progressively increased, rheumatologists have been able to expand the spectrum of 485 monogenic inborn errors of immunity (IEIs), especially primary immunoregulatory diseases (PIRDs) [6–8]. For instance, the genotype-first approach allowed for the identification of VEXAS syndrome (vacuoles, E1 enzyme, X-linked, autoinflammatory, somatic syndrome), a late-onset IEI [9], which, in turn, was found to be more prevalent than expected [10]. Emerging monogenic diseases are expanding the phenotypic spectrum of rheumatic diseases, disrupting conventional paradigms and underscoring the relevance of noncoding genetic variations [1].

Genetics analysis has resulted in the reclassification of some subgroups of rheumatic diseases according to similarities in molecular pathway activation [11]. Furthermore, the post-NGS era has driven our interest in molecular diagnosis as an avenue for targeted therapy, family counseling, a deeper understanding of the pathophysiology of rheumatic diseases, and the exclusion of other mimicking conditions. Despite the progress in molecular diagnostics, many practicing physicians often struggle to stay up to date on various platforms, especially regarding genetic test interpretation of uncertain or ambiguous cases. In addition, the molecular diagnosis of 60–70% of autoinflammatory diseases (AIDs), neuromuscular disorders, heritable connective tissue diseases and monogenic bone diseases has not yet been elucidated, and variants of uncertain significance (VUS) pose a challenge to these unmet demands in the coming decades [12, 13]. This article aims to provide an overview of the clinical indications and interpretations of comprehensive genetic testing for rheumatologists.

## Back to basics: rediscovering the genetic universe

Basic genetic concepts are fundamental for broadly understanding the main clinical indications, interpreting the results and determining differences in genetic sequencing tests. The human genome is composed of 3 billion base pairs (bp) of DNA in 22 pairs of autosomal chromosomes and 1 pair of sex chromosomes that are responsible for our entire structural and functional framework encoded by approximately 20,000 genes. Our genetic material is composed of a specific sequence of purine (adenine, guanine) or pyrimidine (thymine, cytosine) nitrogenous bases that undergo histone modifications (mainly acetylation and methylation), which regulate gene expression [14].

The transcription process initiates the cascade of events guiding targeted protein production. Ribonucleic acid (RNA) polymerase assumes a crucial role, first identifying the exact location for the transcriptional complex to assemble and then catalyzing the synthesis of RNA from DNA, culminating in single-stranded messenger RNA (mRNA) transcription [15]. The mRNA may be subsequently regulated in multiple stages by posttranscriptional events that may modulate gene expression [16]. Each gene encodes a specific protein, but only the exonic regions contain the sequences necessary for the translation process. The introns are removed through spliceosome-mediated cleavage shortly after transcription to form mature mRNAs [17]. Several other subtypes of RNA, such as microRNAs (miRNAs), inhibitory RNAs (long noncoding RNAs [lncRNAs]), and small interfering RNAs (siRNAs), can also modulate protein synthesis at the nuclear level [15, 16].

**Fig. 1:**
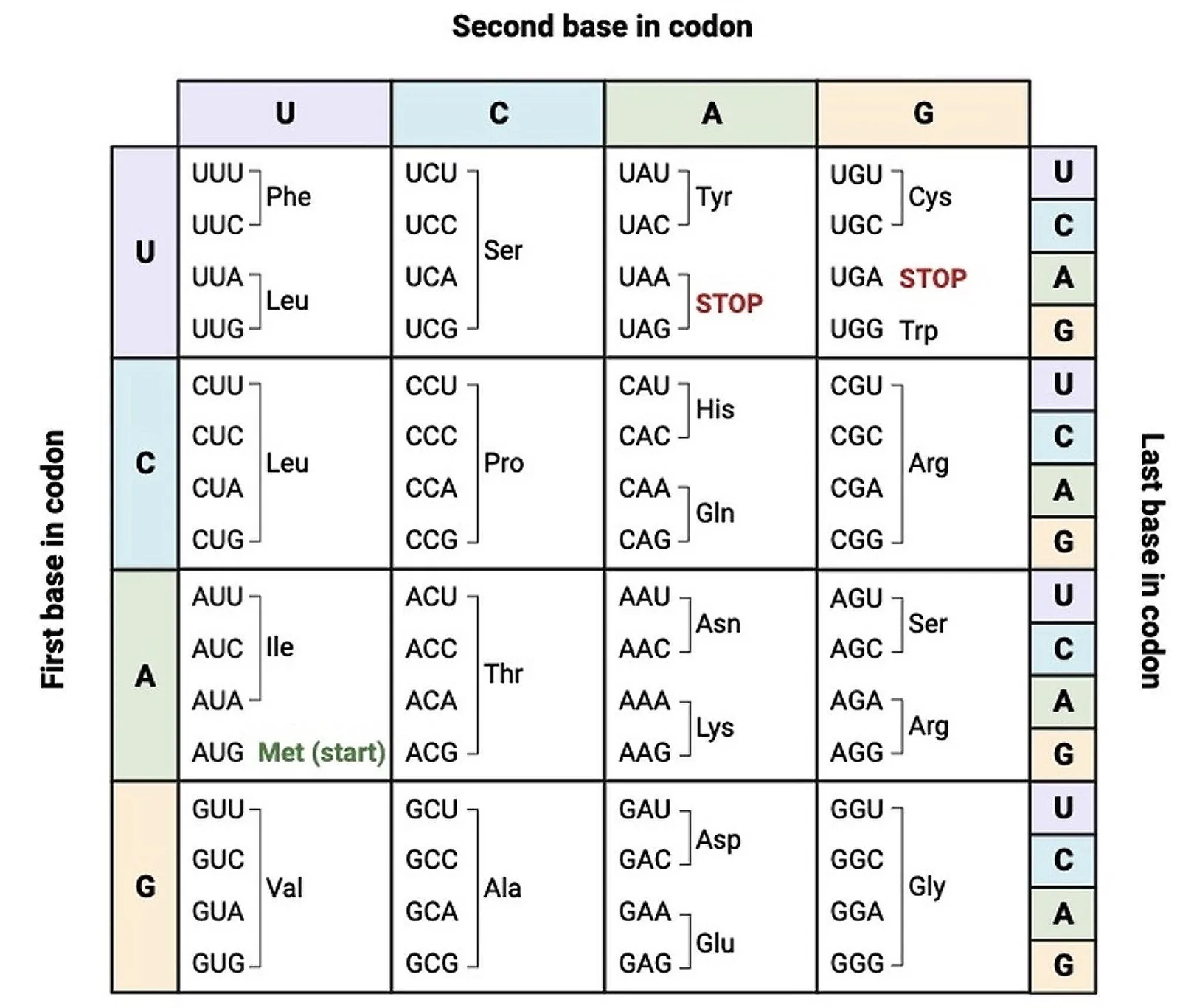
Classification of codons according to the genetic code. *A* adenine, *Ala* alanine, *Arg* arginine, *Asn* asparagine, *Asp* aspartic acid, *C* cytosine, *Cys* cysteine, *G* guanine, *Glu* glutamic acid, *Gln* glutamine, *Gly* glycine, *His* histidine, *Ile* isoleucine, *Leu* leucine, *Lys* lysine, *Met* methionine, *Phe* phenylalanine, *Pro* proline, *Ser* serine, *START* start codon, *STOP* stop codon, *Thr* threonine, *Trp* tryptophan, *Tyr* tyrosine, *U* uracil, *Val* valine. Created in BioRender.com

**Fig. 2:**
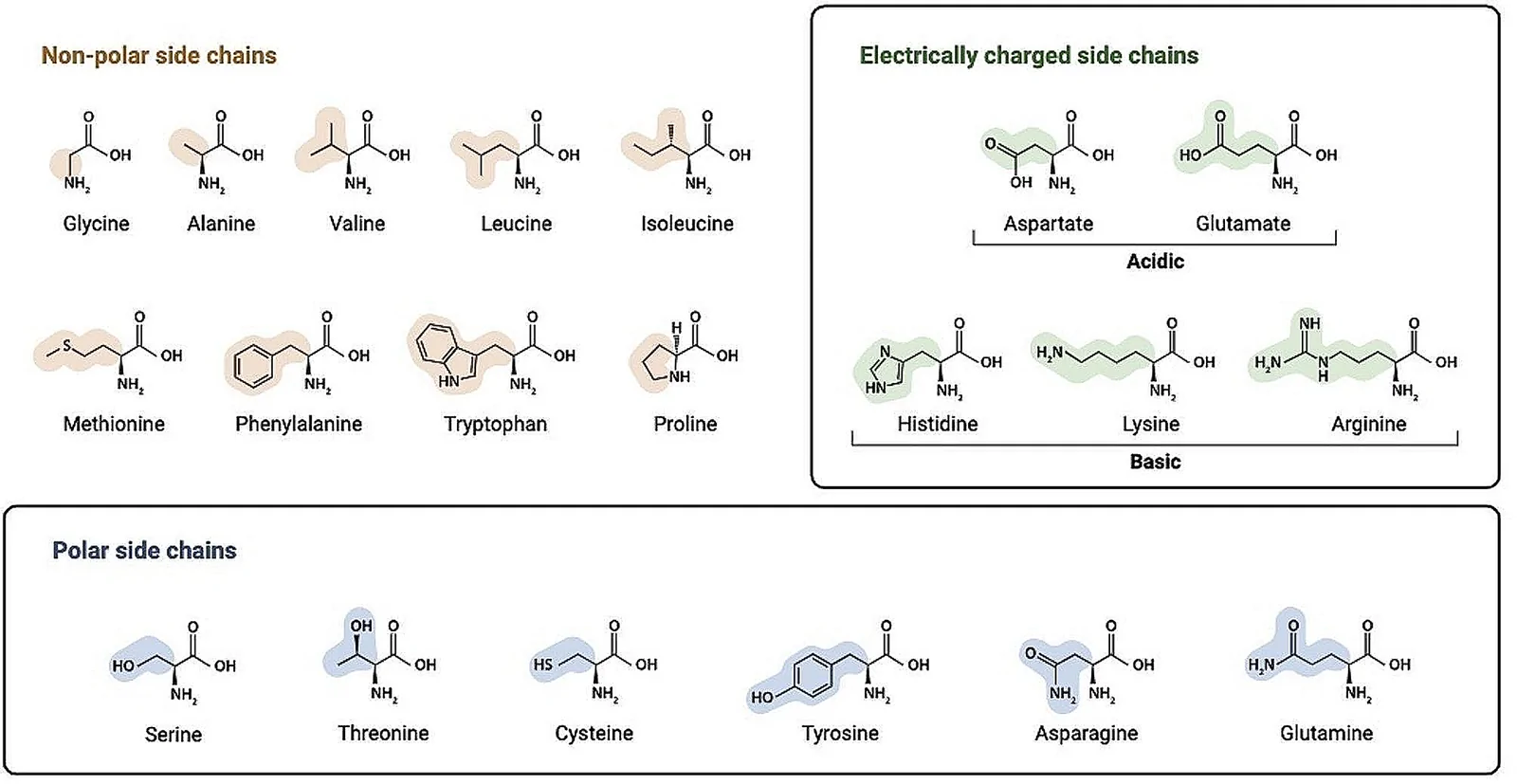
Classification of essential and nonessential amino acids. The figure illustrates nonpolar, polar, and electrically charged amino acids, such as acidic amino acids (negatively charged) and basic amino acids (positively charged). Created in BioRender.com

**Fig. 3:**
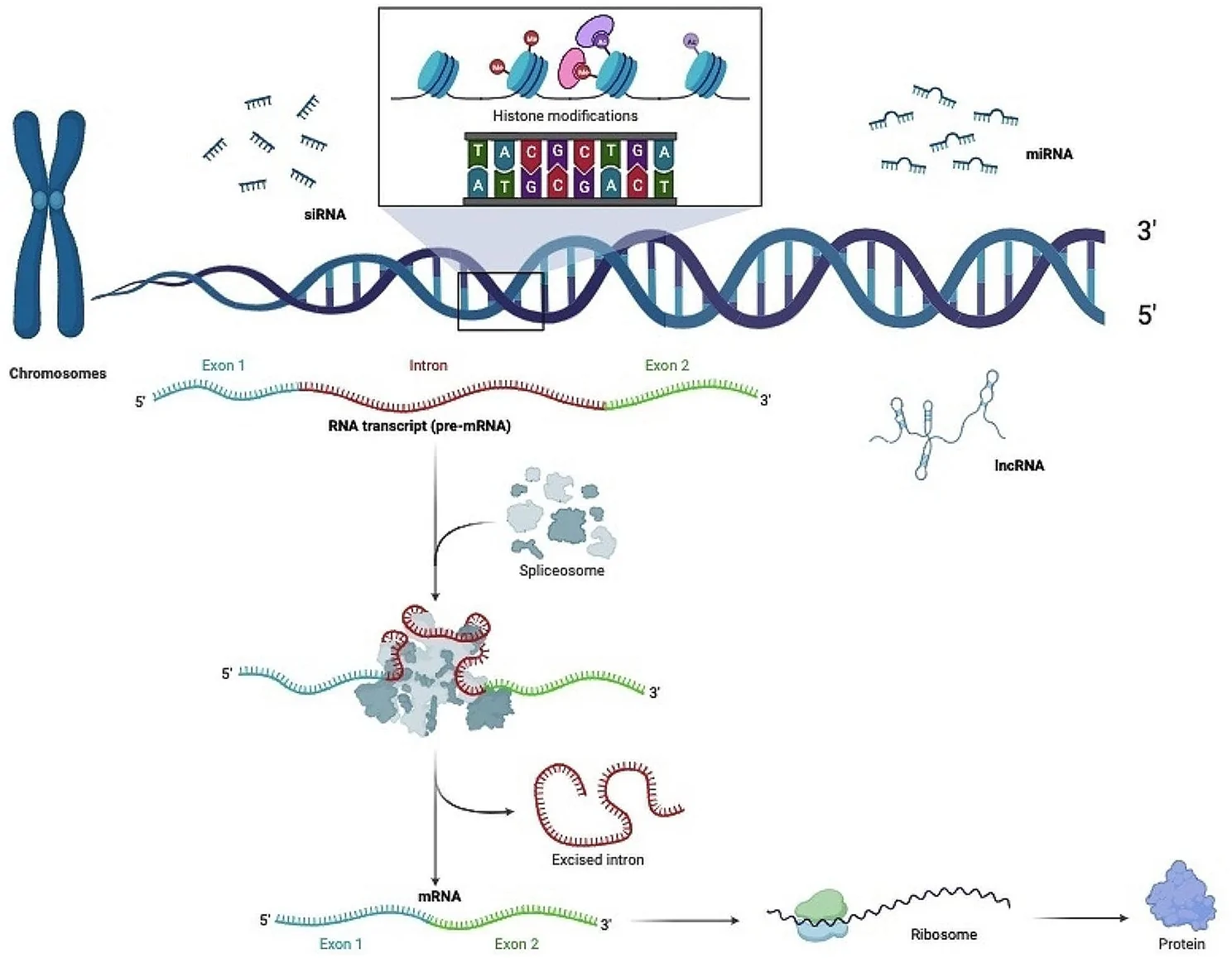
Chromosomes are structures that contain genes, and their genetic material is tightly coiled. Nitrogenous bases (cytosine, guanine, thymine, and adenine) form the fundamental components of each codon (a sequence of three nitrogenous bases) that can undergo epigenetic modifications, such as histone acetylation (exposure of genetic material to transcription factors) or methylation (nitrogenous bases encrypted to external factors). Various factors, such as siRNAs, miRNAs, and IncRNAs, can either enhance gene expression or inhibit gene production. Each gene encodes a single specific protein and undergoes multiple transcription stages in which a set of ribonucleoproteins, known as the spliceosome, degrades the noncoding regions (introns) and retains only the coding regions (exons) during mRNA synthesis. Created in BioRender.com. *A* adenine, *C* cytosine, *G* guanine, *miRNA* microRNA (regulates gene expression), *lncRNA* long noncoding RNA (regulates gene expression), *mRNA* messenger RNA (encodes proteins), *siRNA* small interfering RNA (silencing gene expression), *T* thymine

Proteins are synthesized when a mature mRNA transcript is transported to the cytoplasm and undergoes translation by ribosomes in the endoplasmic reticulum. The base sequence of these coding regions is deciphered by the ribosomal machinery in informational units of three bases, known as codons. Each codon (Fig. 1) either encodes a specific amino acid (Fig. 2) or performs a regulatory function, such as initiating or terminating protein chain synthesis. Aminoacyl transport RNA (tRNA), often referred to as charged tRNA, is an RNA molecule that carries a specific amino acid and possesses an anticodon sequence that is complementary to the mRNA codon [15, 16]. Figure 3 succinctly illustrates this pathophysiological process.

In a gene locus, each DNA copy carries a distinct sequence referred to as an allele. At autosomal loci, there are two alleles, each inherited from a different parent. Autosomal recessive diseases require alterations in both alleles, occurring either in homozygosity (both alleles carrying the same variant) or compound heterozygosity (each allele carrying different variants). Conversely, autosomal dominant diseases manifest with alterations in a single allele carrying a heterozygous variant. In females, who have two X chromosomes, all loci within the X chromosome have two alleles. In contrast, in males, who have one X chromosome and one Y chromosome, each X and Y chromosome locus has only one allele. X-linked diseases may manifest in males when alterations occur in the unique X chromosome allele (hemizygosity) or, occasionally, in females when one of the X chromosome alleles is altered (heterozygosity) [18].

De novo genetic variants do not exist in parents and are frequently associated with autosomal dominant disorders when one mutated allele is sufficient to induce the clinical pathological phenotype. Approximately 80% of de novo variants originate on the paternal allele and are associated with an advanced paternal age at conception [18]. All types of genetic variants can be categorized as constitutional (previously referred to as “germline”), when they are present in all cells, or as somatic, when they occur either shortly after the initial zygotic divisions (early-onset mosaicism) or in adulthood (late-onset mosaicism) [19].

The Goldilocks effect describes the paradigm of achieving the ‘just right’ balance of gene-encoded protein function, especially with respect to wild-type variants [20]. Hypermorphic variants, known as gain-of-function variants, increase protein function. Hypomorphic variants are associated with different mechanisms, including partial function caused by haploinsufficiency (when a single variant reduces the overall protein production by approximately half without interfering with healthy allele transcription), dominant negative effects (when a single variant interferes with healthy allele function), and complete loss of function (often associated with autosomal recessive inheritance) [21].

There are several challenges in pedigree analysis and interpretation of the genetic basis of a condition within a family. First, incomplete penetrance, in which not every individual harboring the same pathogenic variant will consistently exhibit a clearly defined clinical phenotype, can occur. Second, clinical expressivity is variable, as several diseases exhibit substantial diversity in the manifestation of clinical features associated with a particular genetic variant.

## Classification of gene variants

In recent decades, some genetic nomenclature has caused confusion due to misleading concepts. Traditionally, a “mutation” was defined as a permanent alteration in the nucleotide sequence, typically occurring at a frequency of less than 1%, while a “polymorphism” was defined as a variant with a frequency greater than 1%. To enhance clarity and align with recommendations from the American College of Medical Genetics and Genomics (ACMG), replacing these terms with more standardized and precise terminology is advisable. The ACMG recommends employing the term “variant” alongside the following modifiers: (i) pathogenic, (ii) likely pathogenic, (iii) VUS, (iv) likely benign, or (v) benign. This revised terminology better allows for clear and consistent communication in medical genetics, ensuring that the significance of genetic variations is conveyed accurately [22].

Variations may also be divided in the manner that they occur. Structurally, they can be classified as silent, missense, nonsense, frameshift, splicing, or reversion genetic variants. A silent variant is a variant in which a single nitrogenous base is substituted for another, resulting in a change in the codon but not affecting the encoded amino acid. Consequently, the protein remains unaltered. These variants can occur because different sequences of nitrogenous bases can encode the same amino acid. These variants are usually not pathogenic and are commonly referred to as “synonymous” [23].

Missense variants occur when a single nucleotide in the DNA code changes, replacing one amino acid with another within a protein. These variants may maintain normal protein expression or result in protein dysfunction or instability, potentially causing disease [24]. While most missense variants are often classified as VUS, in silico tools are valuable in predicting pathogenicity. Additionally, functional studies, posttranslational modifications, protein‒protein interactions, and protein 3D features in database software contribute to a comprehensive assessment [25].

A nonsense variant occurs when a single nitrogenous base change transforms an amino acid codon into a stop codon leading a protein to terminate or end its translation earlier than expected. This change halts protein synthesis, typically resulting in an unstable and dysfunctional truncated protein, usually with loss-of-function characteristics. The position of nonsense variants plays an important role in protein function and predicting phenotype severity [26].

INDEL (insertion-deletion) are variants caused by insertions or deletions of nitrogenous bases. If a base pair insertion or deletion occurs in a multiple of three starting from the first “wobble” nucleotide, which represents the creation or disappearance of balanced codons, an “in-frame” variant is created; therefore, the protein sequence is altered exclusively at this position. However, if the number of inserted or deleted nucleotides is not a multiple of three or does not start at the beginning of a codon, the reading frame will be disrupted from that point on, causing a “frameshift” that completely changes the sequence and often creates a profoundly altered, unstable, dysfunctional protein. In these cases, a stop codon typically occurs within fewer than 100 nucleotides, resulting in prematurely truncated protein products [27].

Splicing variants are caused by alterations in the genetic rearrangement between exons and introns during mRNA formation. These variants lead to the production of modified mature mRNAs. Variants occurring at the first and second positions before the beginning of a new exon or at the first and second positions after the end of an exon, the so-called canonical splicing sites, are most frequently linked to splicing alterations. In most canonical splice site variants, the mRNA is either destroyed or translated into unstable and/or dysfunctional proteins [28, 29].

Reversion variants occur in exceptional cases of somatic mosaicism, triggered by the restoration of an inherited pathogenic variant to a normal state. In reversion mosaicism, the reversion variant serves to partially or fully reinstate the effect of the primary disease-causing variant. The most common and simplest type of reversion is a true back variant, which refers to the reversal of the constitutional variant site to the wild-type sequence [30]. The primary genetic variant types and their impact on the gene products are detailed in Table 1.

**Table 1:** Primary genetic variant types. Herein, the wild-type gene has a short nucleotide sequence that encodes a protein with an amino acid sequence that reads “I am rheumatologist”. Different variants disrupt this sequence in different ways. The underlined letters correspond to variants of either nucleotides within the codon regions or amino acids

Variant	Example
Wild-type protein	Protein: I A M R H E U M A T O L O G I S T *
	Nucleotide: AAT ATG TAC AAC GCA ATC GAA ATA AAC TAC GAC TGA GTA TGA CCC ATG CGC GAC TAG
Nonsense (stop codon)	Protein: I A M R H E U M *
	Nucleotide: AAT ATG TAC AAC GCA ATC GAA ATA AAC TA**G** GAC TGA GTA TGA CCC ATG CGC GAC TAG
	Example: p.C135X classified as PSMB8 pathogenic variant causing CANDLE syndrome. Changing cysteine (C) at amino acid 135 produce a stop codon [31].
Missense (point mutation)	Protein: I A M R **M** E U M A T O L O G I S T *
	Nucleotide: AAT ATG TAC AAC GCA A**A**C GAA ATA AAC TAC GAC TGA GTA TGA CCC ATG CGC GAC TAG
	Example: p.(Met694Val) is classified as a pathogenic variant in the MEFV gene, causing FMF. This variant involves the substitution of methionine (Met) with valine (Val) at position 694, disrupting the pyrin protein [32].
Deletion (in-frame)	Protein: I A M R H E U M A T O G I S T *
	Nucleotide: AAT ATG TAC AAC GCA ATC GAA ATA AAC TAC GAC TGA --- --- CCC ATG CGC GAC TAG
	Example: p.(Ala125Arg176del) is classified as a pathogenic variant in the MVK gene, causing MKD. This variant involves a deletion of 51 nucleotides between alanine (Ala) 125 and Arginine (Arg) 176, resulting in truncation of the MVK protein [33].
Deletion (frameshift)	Protein: I A M R H E U M A T O K B U D…
	Nucleotide: AAT ATG TAC AAC GCA ATC GAA ATA AAC TAC GAC TGA --- --- --CAT GCG CGA CTA G…
	Example: p.(His91Leufs*12) classified as ADA 2 pathogenic variant causing DADA-2. Frameshift deletion at position 91, resulting in the substitution of histidine (His) into leucine (Leu) and the creation of a premature stop codon 12 nucleotides downstream [34].
Splicing	Protein: * I A M R H E U M A T O L O G I S T
	Nucleotide: A**G**T ATG TAC AAC GCA ATC GAA ATA AAC TAC GAC TGA GTA TGA CCC ATG CGC GAC TAG
	Example: c.671+5G>A classified as IKBKG pathogenic variant causing NDAS syndrome. Guanine (G) is substituted by adenine (A) at the fifth nucleotide after position 671 in the DNA coding sequence. This intronic variant disrupts the coding region, resulting in a protein that is absent [35].
Reversion	Germline variant
	Protein: I A M R **M** E U M A T O L O G I S T *
	Nucleotide: AAT ATG TAC AAC GCA A**A**C GAA ATA AAC TAC GAC TGA GTA TGA CCC ATG CGC GAC TAG
	Spontaneous somatic variant
	Protein: I A M R **H** E U M A T O L O G I S T *
	Nucleotide: AAT ATG TAC AAC GCA A**T**C GAA ATA AAC TAC GAC TGA GTA TGA CCC ATG CGC GAC TAG
	Example: c.995C>T is classified as a pathogenic variant in the WASP gene, causing WASP syndrome. In the germline, the variant results in a cytosine (C) being substituted by thymine (T) at position 995 of the coding DNA sequence, leading to a disrupted protein. However, a spontaneous somatic variant, 995C>T>C, substitutes thymine back to cytosine, restoring the wild-type protein [36].

*ADA 2* adenosine deaminase 2, *CANDLE* chronic atypical neutrophilic dermatosis with lipodystrophy and elevated temperature, *DADA2* deficiency of adenosine deaminase 2, *FMF* familiar mediterranean fever, *IKBKG* Inhibitor of nuclear factor kappa B kinase regulatory subunit gamma, *MEFV* pyrin, *MKD* mevalonate kinase deficiency, *MVK* mevalonate kinase, *NDAS* NEMO-deleted exon 5 autoinflammatory syndrome, *PSMB8* proteasome 20S subunit beta 8, *WASP* wiskott-aldrich. Adapted from Torgerson et al. Stiehm’s Immune Deficiencies 2020 [37]

A variety of publicly or commercially available in silico pathogenicity prediction tools can help determine the chance that a sequence variant is harmful. These tools employ diverse computational algorithms to assess the variant’s impact on both nucleotide and amino acid sequences in the protein. It may predict the effect of genetic variants on the structure or function of a protein without conducting functional tests. Several software tools have been designed to assess different variant types [22]. Meta-predictors that utilize machine learning algorithms and integrate different sources of data, such as REVEL (rare exome variant ensemble learner) and BeyesDel, have gained prominence [22, 38, 39]. Caution is advised when using these prediction tools, and one should refrain from relying solely on them to make clinical decisions. The term “damaging” does not necessarily imply “pathogenic”, since a variant that damages a gene may not be inherently harmful to an individual’s health [40].

An increasing number of variants are being deposited in population databases. These databases play an essential role in classifying gene variants, assessing their pathogenicity, and aggregating diverse sources of validated articles to predict their population risk of appearance. Table 2 provides examples of key databases that can be valuable in the assessment of gene variants [41–45]. Clinicians can also use these different tools to interpret genetic variations and their connections with phenotypes.

**Table 2:** Population, disease, and sequence databases. These databases are valuable tools for querying identified gene variants, as they can be continually updated with new insights related to population frequency, functional validation studies, and key references from relevant research

Genetic database	Description
ClinVar	A repository of statements regarding the clinical importance of human genetic variations and their connections with phenotypes [44].
http://www.ncbi.nlm.nih.gov/clinvar	
OMIM	A database of human genetic information, including genes and genetic conditions, along with a comprehensive representation of various examples [43].
http://www.omim.org	
gnomAD	A website focused on aggregating and harmonizing both exome and genome sequencing data from a wide variety of large-scale sequencing projects and making summary data available for the wider scientific community [41].
https://gnomad.broadinstitute.org/	
VarSome	A database of search engines, aggregators and impact analysis tools for human genetic variation and a community-driven project aiming at sharing global expertise on human variants [41].
https://varsome.com/	
Infevers	A database gathering updated information on genetic variants responsible for hereditary autoinflammatory diseases [42].
https://infevers.umai-montpellier.fr/	
Human Gene Mutation Database	A database containing annotations for genetic variants published in scientific literature. Access to this database requires a paid subscription [45].
http://www.hgmd.org	

Adapted from Gudmundsson et al. [41–45]

## Types of genetic tests

Clinical genetic testing has become increasingly accessible and cost-effective, providing a variety of techniques with unique advantages and limitations. Sanger sequencing (SS), NGS targeted gene panels, WES, WGS, and chromosomal analyses (CAs) are the primary options for achieving a genetic diagnosis [46, 47]. Physicians should choose the appropriate methodology according to the specific clinical context and other factors, such as the scope of analysis, cost constraints, and the nature of the genetic condition under investigation. Table 3 lists the characteristics, including the strengths and limitations, of each method [12, 48].

**Table 3:** Currently available genetic tests

Category	SS	CA	NGS Panels	WES	WGS
Number of genes	1–10	≅ 20.000	10–300	≅ 20.000	All genome
Data size (gigabytes)	0.01	5–10	<1	5–10	50–200
Estimated cost	US$10–20	US$800–1000	US$200–500	US$800–1000	US$1500–2500
Strengths	• Low cost • Fast result time • >99% accuracy	• Detection of CNVs • Detection of absence of heterozygosity	• Simultaneous analyses of multiple genes • Detection of lower-grade mosaicism	• Covers all the coding regions of the genome • Detection of high-grade mosaicism • Data reanalysis • Detection of large CNVs	• Covers the whole genome • Best for small and large CNV identification • Detection of high-grade mosaicism • Data reanalysis
Weaknesses	• Limited coverage of sequences • CNV identification	• CNVs of uncertain significance • Balanced structural variants • CNVs smaller than 100 kb	• Poor coverage of sequences shared with pseudogenes • CNV identification • Inability to store data for reanalysis	• High cost • Identification of small CNVs • High number of VUS identified • Inability to detect intronic variants	• High cost • High number of VUS identified • Storage limitations

*CA* chromosomal analysis, *CNV* copy number variation, *NGS* next-generation sequencing, *SS* Sanger sequencing, *WES* whole-exome sequencing, *WGS* whole-genome sequencing

Adapted from Schnappauf et al. and Chinn et al. [12, 48]

## Sanger sequencing (SS)

In the 1970s, a method originally described by Frederick Sanger was the gold standard for identifying single-gene disorders [49]. SS involves a manual analysis process in which nucleotide pairs are examined exon by exon, which limits automation. SS involves the construction of specific DNA primers for each region of interest that direct in vitro DNA replication by DNA polymerase. Chain-terminating dideoxynucleotides are randomly incorporated in this process, generating DNA fragments of different sizes that are subsequently analyzed using gel electrophoresis and, more recently, capillary electrophoresis to detect genetic variants [50]. SS is highly accurate but is limited to specific genes and may be too time-consuming and costly for broader analysis. Notably, this method may fail to detect copy number variations (CNVs), such as microdeletions or microduplications, or somatic variants characterized by a low variant allele frequency (VAF) [48].

## Next-generation sequencing targeted gene panels

**Fig. 4:**
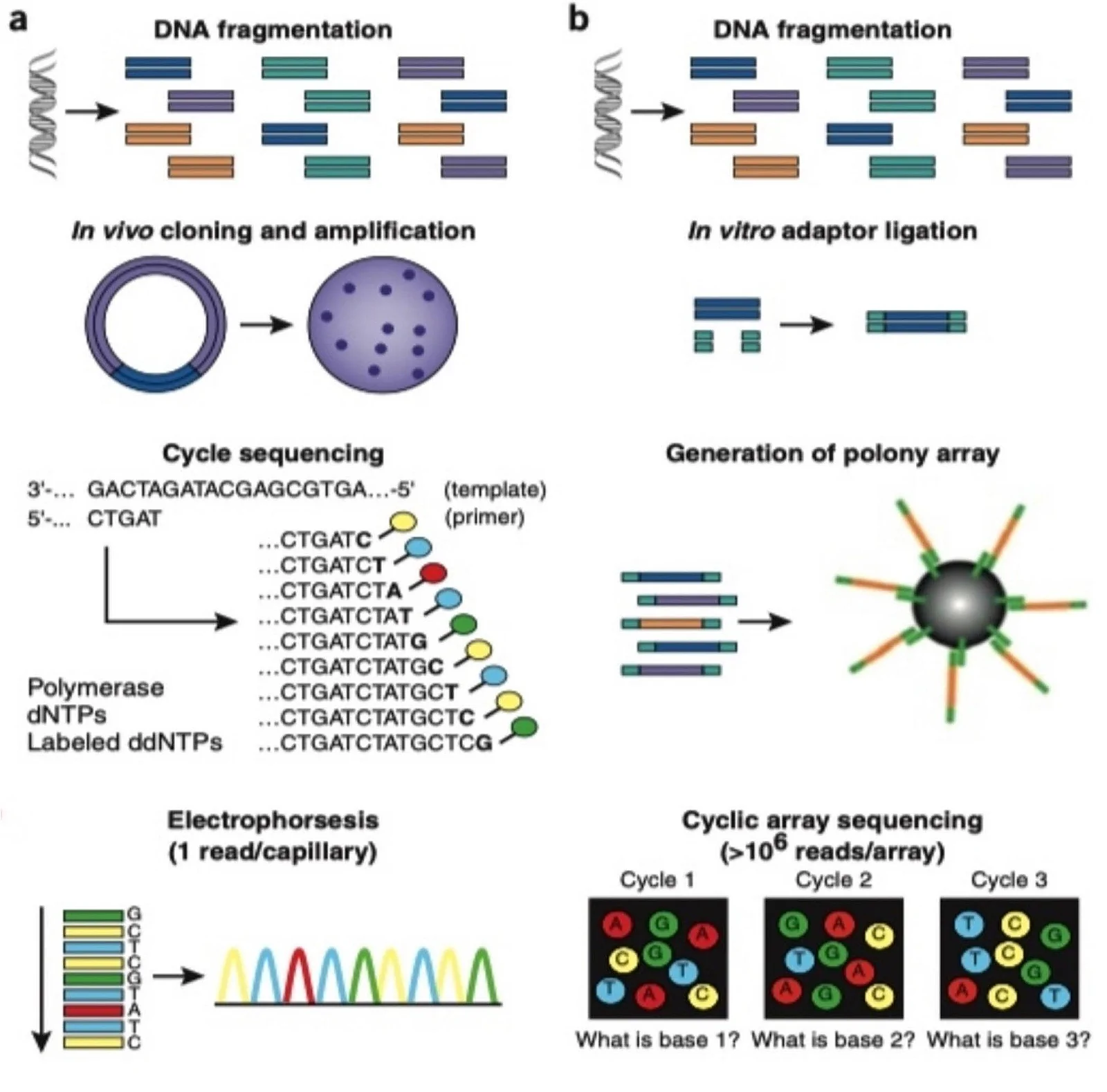
General methods of DNA sequencing encompass both traditional Sanger sequencing (**a**) and next-generation sequencing (**b**). **a** Sanger sequencing involves PCR amplification of genomic DNA fragments containing gene coding regions, followed by sequencing using labeled di-deoxy nucleotides. The amplified fragments are separated by capillary electrophoresis to generate a chromatogram, facilitating sequence determination. **b** In contrast, next-generation sequencing techniques, such as shotgun sequencing, begin with fragmentation of genomic DNA by sonication or enzymatic methods. Linkers are added to DNA fragments, which are then immobilized on solid supports like beads. Emulsion PCR amplifies these fragments, with labeled deoxy nucleotides flowing into reaction chambers containing polymerase and reaction buffers. Incorporation of each nucleotide emits detectable light or ions, allowing real-time sequencing. This approach enables high-throughput sequencing via cyclic-array methods, where millions of immobilized PCR colonies (“polonies”) facilitate parallel processing of sequencing reactions. Imaging-based detection of fluorescent labels during enzymatic extensions enables simultaneous acquisition of sequencing data across all features, resulting in contiguous sequencing reads for each array feature. Figure modified from Shendure and Torgerson et al. [37, 50]

Unlike in SS, multiple genes can be simultaneously analyzed in NGS using an automated approach. When several potential monogenic causes fit a well-established phenotype, the use of NGS becomes necessary, because it is cost effective and faster. Currently, there are targeted panels, which detect fewer genes, and expanded panels; the choice of panel depends on the specific cause under investigation [51]. There are several NGS platforms, and each platform has unique steps for sample preparation, library elaboration and sequencing. DNA extraction from a biological sample followed by fragmentation generally constitutes the initial step. Next, the genomic regions of interest are isolated and enriched (a step often referred to as capture); for targeted gene panels, a limited subset of genes, varying from a few to hundreds, may be enriched. Linkers are affixed to the termini of DNA fragments, and these fragments are tethered to a solid support, commonly a bead, where they are typically amplified through an emulsion polymerase chain reaction (PCR) method. In this process, the information derived from nitrogenous bases is transformed into binary sequences, and the outcome is computationally analyzed, often resulting in a substantial volume of data [37]. Figure 4 provides a concise overview of the key differences between SS and NGS methods [37, 50].

Some advantages of targeted gene panels include the ability to study multiple regions and genes of the genome (more than can be studied with SS) at a relatively low cost (generally lower than that of WES or WGS). Moreover, mosaicisms with lower VAFs and CNVs, such as microdeletions and microduplications, may be detected. However, pseudogenes may lead to misinterpretation of variant results [48].

## Whole-exome sequencing

WES covers the coding regions of the genome (exons), making it an automated method that generates a substantial volume of data for bioinformatics analysis. When the clinical phenotype is nonspecific for a single causative gene, a defined group of genes or even a unique metabolic pathway, WES has been shown to be a pivotal strategy for investigating a wide spectrum of genetic disorders. The steps of WES steps are very similar to those of NGS targeted gene panels, as discussed above. The isolation and fragmentation of genomic DNA are followed by the addition of oligonucleotide adapters. Fragmented adapter-ligated DNA libraries necessitate an additional positive selection capture step to prevent off-target sequencing of noncoding genome regions. The ideal result is equal capture of all exome regions; however, enrichment tends to be uneven [52]. As most known monogenic defects are located within coding regions, WES is a valuable and relatively accessible diagnostic tool that is more affordable than WGS. Nevertheless, due to the large amount of data, VUS frequently emerge, posing a challenge in interpretation. WES allows for the study of large CNVs and high-grade mosaicism, despite its limitations in identifying relevant intronic variants, low-grade mosaicisms and small CNVs [53].

## Whole-genome sequencing

WGS covers most of the genome, encompassing coding and noncoding regions. However, its widespread availability is limited by its high cost and the relatively low number of known intronic pathogenic variants; therefore, this application is currently indicated for clinical research purposes [54]. The WGS methodology closely resembles that of WES, with the notable exception of the absence of an exome enrichment step. The process involves DNA fragmentation, attachment of linker sequences, and subsequent massively parallel sequencing. WGS technologies can be categorized on the basis of their capacity to read short sequences (< 1 kilobase) versus long sequences (> 1 kilobase). Long-read sequencing, despite its relative clinical unavailability, shows promise for mitigating DNA fragmentation, offering deeper reads without sacrificing any nucleotide bases during the process [55, 56]. WGS is more effective at detecting CNVs than WES is and has the potential to identify novel disease-causing gene variants. However, due to the “big data” analysis involved, storing raw data for future reanalysis is a challenge. Moreover, the high cost, coupled with the significant volume of VUS and unknown intronic variants, currently presents substantial hurdles to larger routine laboratory use [56].

## Chromosomal analyses

Various techniques can be employed to assess CAs, such as karyotyping, microarray analysis, and fluorescence in situ hybridization (FISH). While karyotyping can reveal large deletions, duplications, translocations and inversions, it is limited in the identification of microdeletions, microduplications, or smaller rearrangements that may be detectable only by microarray or FISH [57]. CA techniques enable the identification of chromosomal losses and gains and are recommended as first-tier approaches, particularly for syndromic phenotypes characterized by dysmorphic features, congenital malformations, failure to thrive or neurodevelopmental disorders [58]. The generally fast turnaround time and lower cost of these methods compared to WES or WGS represent significant advantages. However, confirming CNVs of uncertain significance can be challenging, and the detection of unbalanced chromosomal rearrangements smaller than 100 kb may be challenging [59].

## Practical approach to order genetic tests

Early genetic sequencing in selected patients, whether through preestablished multigene panels or WES, is indicated for diagnosing monogenic diseases. Given the extensive clinical spectrum of genetic diseases of interest in rheumatology, it is challenging to establish universal guidelines and warning signs. The Jeffrey Modell Foundation has devised several warning indicators for IEIs that can aid in matching a specific phenotype with its related molecular diagnosis (Table 4) [60, 61].

**Table 4:** IEI warning signs from the Jeffrey Modell Foundation: clues for diagnosis

**1. Recurrent infections:** severe, persistent, or atypical infections; difficulties in controlling EBV infections; bronchiectasis; and infections not explained by known factors such as COPD, asthma, HIV, diabetes, hyposplenism, asplenia, hematological malignancy, or treatments involving glucocorticoids or DMARDs
**2. Rheumatic disorders:** early-onset JIA; sarcoidosis; enteropathic arthritis; benign lymphoproliferation; and extra-articular conditions such as ILD, IBD, and pustular psoriasis
**3. Malignancies:** lymphoma, gastric cancer, and NMSC
**4. Family history:** PID, rheumatic disorders, IBD or related enteropathies, and lymphoma

*COPD* chronic obstructive pulmonary disease, *DMARD* disease-modifying antirheumatic drug, *EBV* Epstein–Barr virus, *HIV* human immunodeficiency virus, *IBD* inflammatory bowel disease, *IEI* inborn error of immunity, *ILD* interstitial lung disease, *JIA* juvenile idiopathic arthritis, *NMSC* nonmelanoma skin cancer, *PID* primary immunodeficiency disease [60, 61]

In the pre-NGS era, a molecular assay targeting an individual gene typically represented the conclusive phase of the diagnostic process, after clinical, laboratory, and histological assessments aimed at delineating the most likely diagnosis. As NGS becomes more integrated into routine clinical diagnostics, sequencing methods are frequently employed at an earlier stage, immediately following a thorough clinical evaluation [3].

In addition, as the costs of genetic tests have decreased, there has been a continuous increase in the identification of new variants responsible for novel Mendelian diseases [62]. Targeted gene panels focusing on heritable extracellular matrix diseases and thoracic aortic aneurysms played a pivotal role in elucidating the genetic underpinnings of autosomal dominant diseases, such as Marfan syndrome, vascular Ehlers–Danlos syndrome, and Loeys–Dietz syndrome, that were previously considered extremely rare and were revealed to be notably prevalent. Molecular diagnostics serve as a valuable tool for distinguishing these conditions from vasculitis mimickers, aiding in accurate diagnosis and preventing unnecessary immunosuppression [63]. Moreover, with the increased availability of genetic panels, polymyositis has become an increasingly rare entity due to the recognition of inherited neuromuscular diseases [64].

The identification of monogenic bone diseases can be an integral part of investigating unexplained reductions or increases in bone mineral density, bone mineralization or bone turnover. Genetic sequencing is fundamental for corroborating the clinical diagnoses of patients with *osteogenesis imperfecta*, juvenile Paget disease, or *fibrodysplasia ossificans progressiva* [65].

**Fig. 5:**
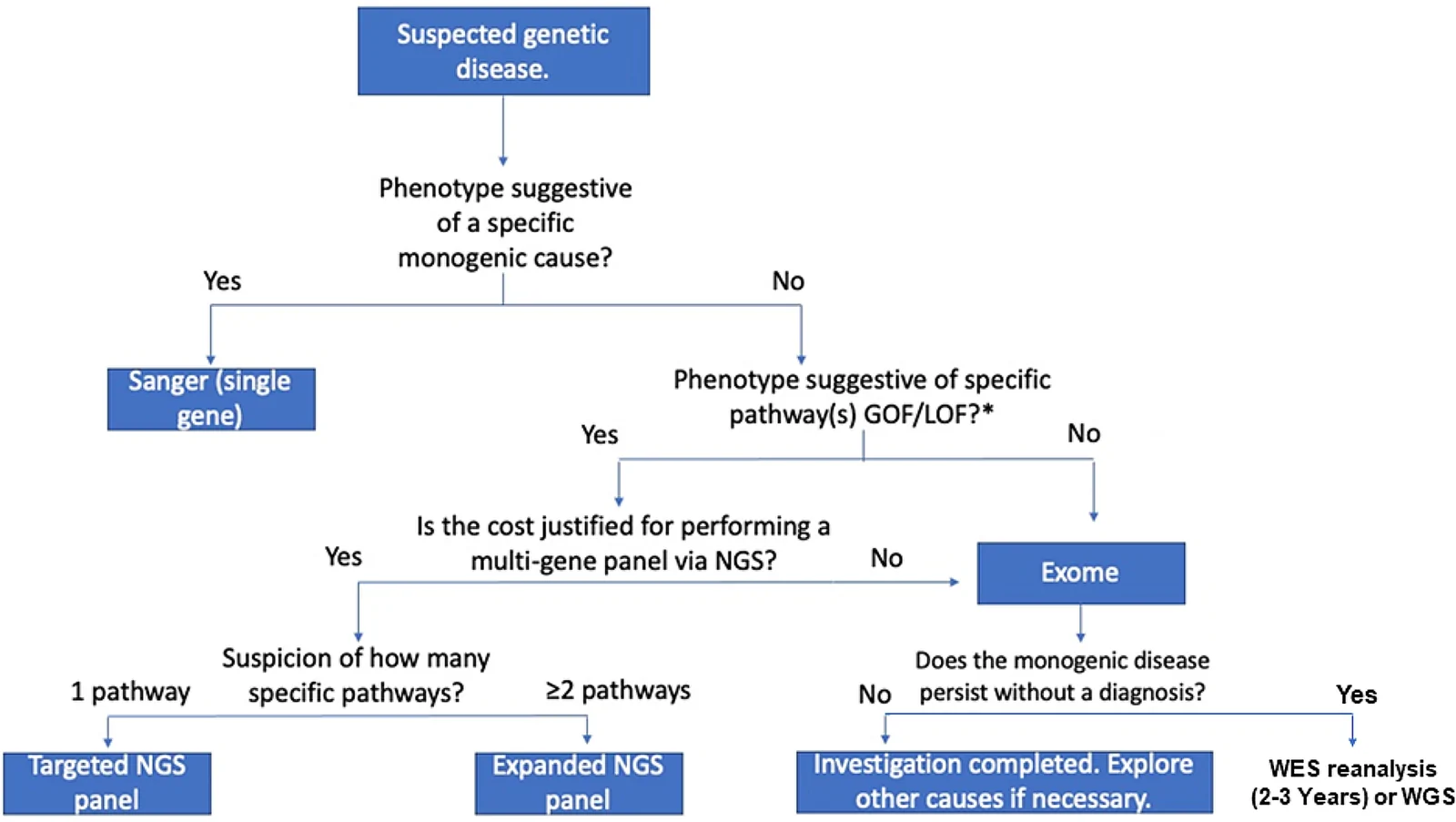
Suggested algorithm for molecular investigation. *GOF* gain-of-function, *LOF* loss-of-function, *NGS* next-generation sequencing, *WES* whole-exome sequencing, *WGS* whole-genome sequencing; *metabolic myopathies, muscular dystrophies, congenital bone diseases, heritable connective tissue disorders, or inborn errors of immunity can be considered in the phenotyping

Notably, it is imperative that any genetic sequencing results are consistently interpreted in light of the clinical phenotype and the identified molecular pathway. Figure 5 presents a suggested algorithm for molecular investigation. Numerous parameters must be considered in the classification and interpretation of a variant. Zygosity is a crucial factor, as monoallelic variants can cause autosomal dominant conditions, whereas biallelic variants contribute to recessive disorders. The assessment of pathogenicity hinges upon several facets, including population frequencies of the variants in genome databases, computational and in silico predictions, functional data, segregation analysis within family pedigrees, allelic data, functional insights, and patient phenotype. It is essential to emphasize that these data should not be interpreted in isolation and must always be evaluated within the context of the relevant metabolic pathway [22].

Clinicians should not categorically attribute a VUS as the primary cause of a condition solely based on its apparent clinical relevance, as variant pathogenicity classifications evolve over time, encompassing shifts toward both increased and reduced pathogenicity [66]. Only a small proportion of VUS are likely to ultimately demonstrate pathogenicity upon subsequent evaluation. Unfortunately, the clarification of this uncertainty is often a protracted process. In silico predictors, proximity to previously described hotspots, functional investigations, and parental segregation studies can provide valuable assistance in the clinical decision-making process [67].

One of the most efficacious approaches for obtaining insights into the clinical importance of a VUS or for discerning compound heterozygosity in the *trans* configuration is familial segregation analysis [68]. Segregation analysis, which studies the inheritance pattern of a variant within a family, may be a valuable approach to determine the pathogenicity of variants [69]. Segregation analysis can also aid in the identification of de novo variants, providing stronger evidence for pathogenicity. Additionally, it can facilitate the reclassification of rare variants as benign or likely benign. Hence, this step is pivotal in refining the precise interpretation of genetic findings [70].

## Limitations of genetic analysis

Somatic mosaicism may be an explanation for negative genetic analysis results. In these cases, the tissue or cell containing the cryptic variant may have not been assessed, or the VAF in peripheral blood may be so low that even WES or WGS may lack the requisite sensitivity to detected it. Various NGS panels have endeavored to achieve the detection of variants with progressively lower VAFs, exemplified by the search for UBA1 (ubiquitin like modifier activating enzyme 1) in the context of VEXAS syndrome diagnosis [71] or for the *LEMD3* (LEM domain containing 3), *KRAS* (kirsten rat sarcoma viral oncogene homolog)*, MAP2K1* (mitogen-activated protein kinase kinase 1) and *SMAD3* (SMAD family member 3) genes in melorheostosis [72].

Structural variants (SVs) are a category of genetic alterations exceeding 50 bp in length, with some extending up to several megabases (Mb). This category encompasses various changes, including CNVs, deletions, duplications, insertions, inversions, mobile element insertions (transposons), translocations, and complex rearrangements. Short-read sequencing technologies may not detect some SVs due to their limited precision and accuracy. Emerging techniques employing long-read sequencing (100–300 bp long) are being implemented to bypass the genetic material sonication process and prevent nucleotide base losses using nanopore sequencing [13, 73].

While nucleotide repeats form the basis of 3% of the human genome, certain repeat sequences known as short tandem repeats are associated with specific diseases and may remain undetectable using conventional tools. Another limitation of standard platforms arises from pseudogenes, which are segments of DNA structurally resembling genes but lacking the capacity for protein encoding, introducing several biases in regular analysis [53].

Diseases or variants that have never been previously documented in databases can complicate the management of these conditions, which often manifest as ultrarare diseases. One strategy to overcome this challenge is to periodically reanalyze the genetic sequence. Several groups have shown that reanalyzing raw genomic data can boost diagnostic yields by 5–26% for WES and 4–11% for WGS [13, 74]. Despite the promising advances beyond broad-spectrum genetic sequencing methodologies that will be made in the coming decades, some cases are exceedingly intricate and transcend the scope of the general medical practitioner. In these cases, the pursuit of interdisciplinary collaboration, knowledge exchange with specialists, and referral to molecular diagnostic reference centers seems more appropriate.

## Conclusions and future perspectives

The increasing accessibility of genetic tests enhances diagnostic yield and elucidates the molecular underpinnings of rheumatic diseases [11]. Advances in these techniques have reshaped our understanding of the pathophysiology of rheumatic disorders, especially AIDs, neuromuscular disorders, hereditary extracellular matrix diseases, and monogenic bone diseases.

However, as we rely more on these tools, new challenges emerge, such as interpreting VUS, detecting mosaicism, and identifying SVs. Promising solutions include complementing short-read genome sequencing with RNA sequencing, long-read genome sequencing, metabolomics, proteomics, and DNA methylation profiling. Furthermore, novel functional tests are essential for validating novel genetic variant results [13, 73]. Machine learning can serve as a strategy for gathering extensive datasets encompassing various types of biomarkers to complement genetic sequencing [75, 76].

A significant hindrance in diagnosing rare patients is the cost, given that some genetic tests are still research-based and not yet integrated into healthcare systems. Efforts are required to increase the accuracy and affordability of high-throughput technologies, bridging the diagnostic gap for undiagnosed patients. A delicate balance is imperative when considering the cost-effectiveness of molecular diagnoses and personalized targeted therapies as we progress toward precision medicine [77, 78].

## Data Availability

Data sharing is not applicable to this article, as no datasets were generated or analyzed during the current study.

## Abbreviations

A: Adenine
AID: Autoinflammatory disease
ACMG: American College of Medical Genetics and Genomics
bp: Base pairs
C: Cytosine
CA: Chromosomal analysis
CNV: Copy number variation
COPD: Chronic obstructive pulmonary disease
DMARD: Disease-modifying antirheumatic drug
DNA: Deoxyribonucleic acid
EBV: Epstein–Barr virus
FISH: Fluorescence in situ hybridization
G: Guanine
GOF: Gain-of-function
IBD: Inflammatory bowel disease
IEI: Inborn error of immunity
IncRNA: Long noncoding RNA
ILD: Interstitial lung disease
JIA: Juvenile idiopathic arthritis
LOF: Loss-of-function
Mb: Megabase
mRNA: Messenger RNA
miRNA: MicroRNA
NGS: Next-generation sequencing
NMSC: Nonmelanoma skin cancer
PCR: Polymerase chain reaction
PID: Primary immunodeficiency disease
PIRD: Primary immunoregulatory disease
RNA: Ribonucleic acid
siRNA: Small interfering RNA
SS: Sanger sequencing
T: Thymine
UBA1: Ubiquitin like modifier activating enzyme 1
VAF: Variant allele frequency
VEXAS syndrome: Vacuoles, E1 enzyme, X-linked, autoinflammatory, somatic syndrome
VUS: Variant of undetermined significance
WES: Whole-exome sequencing
WGS: Whole-genome sequencing
